# Effect of a multidisciplinary end-of-life educational intervention on health and social care professionals: A cluster randomized controlled trial

**DOI:** 10.1371/journal.pone.0219589

**Published:** 2019-08-19

**Authors:** Sakiko Fukui, Junko Fujita, Sumie Ikezaki, Eiji Nakatani, Mayuko Tsujimura

**Affiliations:** 1 School of Allied Health Science, Department of Nursing, Osaka University, Osaka, Japan; 2 School of Nursing, Juntendo University, Chiba, Japan; 3 School of Nursing, Chiba University, Chiba, Japan; 4 Division of Statistical Analysis, Research Support Center, Shizuoka General Hospital, Shizuoka, Japan; Universita degli Studi Di Cagliari, ITALY

## Abstract

**Background:**

The aging of populations is rapidly accelerating worldwide. Especially, Japan has maintained the highest rate of population aging worldwide. As countermeasures, the Japanese government prioritized the promotion of local comprehensive care systems and collaboration in medical care and social (long-term) care. Development of a system to connect medical and social services in the community is necessary for the increasing older people, especially for the people in the stage of end of life.

**Objective:**

This study aimed to assess the effect of a multidisciplinary end-of-life educational intervention program on confidence in inter-professional collaboration and job satisfaction among health and social care professionals.

**Design:**

a cluster-randomized controlled trial

**Setting/Participants:**

Three professional groups (home care nurses, care managers, and heads of care workers) in an urban area participated in this trial.

**Intervention:**

We implemented a multidisciplinary end-of-life educational intervention program comprising two educational workshops and an educational booklet to support multidisciplinary care for end-of-life patients during the 7-month study period.

**Main outcome measure:**

Confidence in improved interactions among professionals and job satisfaction were assessed with the Face-to-Face Cooperative Confidence Questionnaire and the Minnesota Satisfaction Questionnaire at T1 (before intervention) and T2 (7 months after the intervention).

**Results:**

In total, 291 professionals participated in this study (experimental group n = 156; control group n = 135). Multivariate regression analyses showed significant between-group increases on all of seven subscales in participants’ face-to-face cooperative confidence over the study period; no effect was evident regarding job satisfaction.

**Conclusions:**

A multidisciplinary end-of-life educational intervention program increased confidence in multidisciplinary collaboration among health and social care professionals.

**Trial number:**

UMIN Clinical Trial Registry, Japan UMIN000022772.

## Introduction

The aging of populations is rapidly accelerating worldwide [1]. The number of people aged 65 years or older is projected to grow from an estimated 524 million in 2010 to almost 1.5 billion in 2050 [2]. Therefore, aging nations have growing needs to support the increasing older adult population who need care. Especially, Japan has maintained the highest rate of population aging worldwide since 2004 [3]. As countermeasures, the Japanese government prioritized the promotion of local comprehensive care systems and collaboration in medical care and social (long-term) care [4,5]. In 2025, Japan will become a super-aged nation, in which one-quarter of the population are aged 75 years or older [6]. Thus, development of a system to connect medical and social services in the community is necessary for the increasing older adult population who need medical and social care.

In this context, strengthening inter-professional collaboration among health and social care professionals is important to ensure the medical and welfare system in Japan. In particular, a system to support patients receiving end-of-life care in the community has become increasingly important for this group. For the end-of-life (EOL) stage at home, careful control of symptoms and life support is required for daily changing conditions. Therefore, good collaboration among health- and social-care professionals is essential in providing quality home-based EOL care to increase the chances of dying at home and reducing the symptom burden [7,8]. However, a recent review indicated that no studies have investigated the effect of a multidisciplinary end-of-life intervention program on improving collaborative recognition and attitudes among health and social care professionals [9].

Another major problem in supporting Japan’s aging society is a shortage of medical and social care workers who work in the home care setting. Therefore, it is also important to secure human resources for long-term care in the community. This makes efforts to maintain and increase professional motivation (e.g., elevating job satisfaction) particularly important. A good way to maintain job satisfaction for these professional groups is to strengthen inter-professional collaboration. This study aimed to assess the effect of a multidisciplinary end-of-life educational intervention program on improving confidence in and job satisfaction with inter-professional collaboration among health and social care professionals.

## Materials and methods

### Study design

A cluster randomized controlled trial was implemented in K-city. K-city is located in Kanagawa prefecture, and is an urban area with a population of 1.5 million people. K-city has seven districts, which we randomly divided into the experimental group (four districts) and control group (three districts).

We assessed the efficacy of a multidisciplinary end-of-life intervention program on health/social care professional outcomes: we compared participants from the four districts in the experimental group with those from the remaining three districts (control group). Participants in the experimental group attended two 3-hour programs in the 7-month study period (July 2016 and February 2017). In addition, they used an educational booklet that described how nurses, care managers, and care workers should collaborate to support end-of-life patients at home throughout the study period. Participants assigned to the wait-listed control group were invited to participate in the same program after this study ended.

### Participants

Participants comprised three professional groups engaged in providing home care: care managers, heads of care workers, and home care nurses. In Japan, home care is provided within the long-term-care insurance system. This system has various service providers depending on the type of service. Patients who receive home care have to make contracts with the appropriate agency. The combination of such services can be complex; thus, care managers are responsible for developing care plans by combining services based on each patient’s care level and wishes. The heads of care-worker agencies are responsible for the management of care workers employed in their home-help systems [10]. Almost all care workers are part-time, so the role of heads of care workers is particularly important. Home care nurses belong to visiting nursing stations, which is another type of home care agency. Home care nurses provide medical care under the direction of the doctor in charge at the beginning of home care; thereafter, home care nurses follow care plans created by care managers for the duration of the home-care service [11,12].

### Procedure

First, we chose a city (K-city) in the east of Japan that had professional bodies for home care nurses, care managers, and heads of care workers. To obtain consent to conduct this research, we sought approval for the project from the municipality’s department of long-term care service, as well as the heads of the three bodies of home care services (nurses, care managers, and care workers). Next, we explained on this study at the annual meetings held by each of these bodies. Finally, we requested all agencies in the city to invite their employees to participate in this study by mail. Participants returned a consent form to us by fax.

### Intervention

#### Educational booklet

As part of the intervention program for the experimental group, we developed an educational booklet to enhance end-of-life care collaboration among home care nurses, care managers, and heads of care workers. This booklet was developed based on literature reviews [13–17] and focus group interviews with care managers, heads of care workers, and home care nurses (including our research team members).

The booklet emphasized: (1) mutual respect as professionals who have different expertise, (2) understanding of professionals’ roles, and (3) sharing of information in a timely manner to support end-of-life patients appropriately. The 18-page booklet provided information on how different professional groups and team members could collaborate with each other. The booklet also covered the characteristics and symptoms of patients by dividing the end-of-life stage into eight phases; it made reference to the Gold Standard Framework of the United Kingdom [18]: phase 1, identifying patients who required end-of-life care register; phase 2, teamwork to provide end-of-life care; phase 3, several-months prognosis; phase 4, several-weeks prognosis; phase 5, several-days prognosis; phase 6, 3-days prognosis; phase 7, 24-hours prognosis; and phase 8, several-hours prognosis.

#### Educational workshop: Lecture and group work session

The workshop comprised two sessions (session 1 was a 30-minute lecture; session 2 involved group work to discuss three types of cases with instructors for 150 minutes). In the lecture, participants received an explanation about the aim and content of the booklet to support multidisciplinary collaboration in end-of-life care at home. In the group work session, five or six groups of participants that mixed the three professional groups were formed. One of the present researchers joined each group as a facilitator. Participants discussed the timing and content of information sharing from nurses as medical professionals to the other two social care professional groups, as well as the timing and content of information disseminated from the two social care professional groups to nurses, using a simulation case. Participants performed a role play about sharing information among the three professional groups in a situation where the simulated patient’s condition was exacerbated with reference to the booklet. Throughout the group work session, participants also discussed their feelings in clinical practice. We provided these same workshops at T1 and T2 for participants.

#### Follow-up

During the 7 months after the first workshop, participants were asked to implement the booklet for their end-of-life patients. To encourage participants to use the booklet, participants received newsletters that described concrete application examples based on the booklet every 2 months.

#### Control group

Participants in the control group were asked to provide usual care during the study period, and were asked to respond to the same questionnaire as the experimental group at T1 and T2.

### Theoretical framework

Teamwork is a complex process that requires analysis from various perspectives [19]. Leuts described three levels of teamwork involved in integrating medical and social services [20]. At the linkage level, providers on both the medical and social sides must understand when it is appropriate to communicate information and who is responsible for each patient’s needs. At the coordination level, explicit structures and individual care managers are needed to coordinate services. At the full integration level, facilitates are necessary to support deinstitutionalization of care as well as control of resources. We developed two scales to measure the collaboration competency of home care professionals: (i) a face-to-face cooperative confidence measure for the linkage level [21] and (ii) an interdisciplinary collaborative practice scale to measure the coordination and full integration levels [22].

In addition, we decided to measure job satisfaction in this study. The World Health Organization has found that an inter-professional education elevates a provider’s satisfaction in addition to improving communication among providers [23]. Also, previous studies have shown that job satisfaction is regarded as collective feelings or affective responses associated with a job situation [24] and professionals’ job satisfaction enhances the professional identity [25]. Job satisfaction is correlated with quality of patient care [26]. Considering these previous correlated findings with job satisfaction, we measured job satisfaction as a fundamental aspect for strengthening teamwork.

This manuscript reports the results for the linkage level (face-to-face cooperative confidence) and a fundamental aspect of teamwork (job satisfaction).

### Measures

Baseline data were collected in June 2016 (T1) before the intervention by a questionnaire survey. Seven months later, in January 2017 (T2), follow-up data were collected. Following the same protocol, the control group received the intervention after T2 (as a wait-list control). Data were matched by participants’ ID numbers.

### Outcomes

A Face-to-Face Cooperative Confidence Questionnaire (FCCQ) was developed for home healthcare providers [21]. This face-to-face cooperation level measured the degree of role understanding and communication with each professional group. The scale includes 21 items on seven subscales, and has good internal consistency and reliability [21]. The seven subscales are (i) I can smoothly communicate with medical and social care professionals in other facilities, (ii) I understand other occupational roles of people working in the community, (iii) I know the face, name, and characteristics of people associated with home care in the community, (iv) I have an opportunity to discuss matters with other health care workers in the community, (v) I am connected to community care networks, (vi) I specifically understand community resources, and (vii) A good network is maintained between the hospital and community, such as having a conference before hospital discharge. Responses are on a five-point Likert-type scale from 1 (disagree) to 5 (agree). Higher scores indicate a better relationship (See S1 Appendix).

To measure job satisfaction, we used the short-form Minnesota Satisfaction Questionnaire (MSQ), developed by Weiss et al [27]. The directions for the short-form are identical to those for the long-form [27, 28]. The short-form MSQ comprises 20 items on three scales: intrinsic satisfaction, extrinsic satisfaction, and general satisfaction. The reliability and validity of the short-form MSQ have been established for the English [29] and Japanese versions [30]. Individuals are asked to rate their level of satisfaction with 20 work-related needs on a 5-point scale from “not satisfied” (1) to “extremely satisfied” (5). Responses for all 20 items can be summed to produce a general satisfaction score. We chose 12 intrinsic satisfaction items for this study, because these could be used to measured intrinsic and personal aspects of participants’ job satisfaction.

### Ethical considerations

The Ethics Committee of the Japanese Red Cross University approved this study (No. 2016–003). Participants were informed of the voluntary nature of participation in the study. Return of a completed questionnaire was considered provision of consent to participate. This trial is registered with UMIN Clinical Trial Registry, Japan (UMIN000022772).

### Statistical analysis

As sample size calculation, by referring the data of the sub-scale of the face-to-face cooperation level, we input these parameters; effect size = 0.4, α = 0.05, β = 0.2 and used the software named G*power3.2. As a result, the number of each groups was shown as 100 and the total number became 200. Therefore, we aimed to get the total sample number 300 of 1.5 times, considering the sample’s drop out and deficit of the data.

Demographic and measurement data for participants for each assessment time (T1 and T2) were analyzed using *t*-tests, chi-square tests, or Mann-Whitney *U* tests to assess comparability between the groups. The effects of the intervention on each measure were assessed using multivariate regression analyses to test for differences between the experimental and the control groups over time, after adjusting for variables that showed *p-*values < .10 between groups in the univariate analysis. *P*-values < .05 were considered statistically significant. All analyses were performed using SAS statistical software version 9.4.

## Results

### Study sample

**Fig 1:**
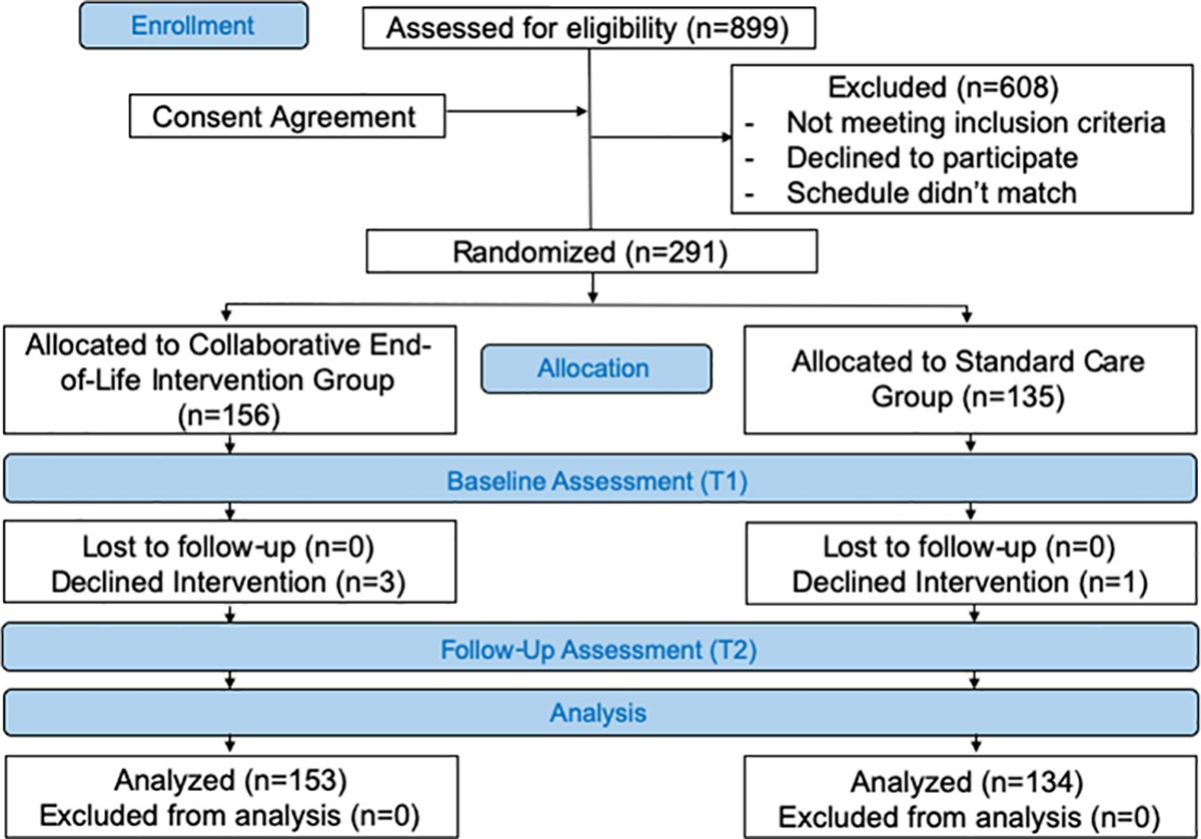
CONSORT 2010 flow diagram.

K-city municipality’s department of long-term care service and the heads of the three relevant professional bodies (home care nurses, care managers, and care workers) consented to participate in the study. Of the 899 eligible participants (99 nurses, 398 care managers, and 402 heads of care workers) who work in K-city, 291 consented to participate in this study and completed the questionnaires at T1 and T2 (64 nurses, 129 care managers, and 98 heads of care workers). The experimental and control groups were formed from homecare nurses (n = 31 and 33), care managers (n = 72 and 57), and head care workers (n = 53 and 45). Throughout the 7-month study duration, three participants of experimental group and one participant of control group were dropped out. The reasons were that two participants moved to other area and quit the job and another two declined because they were busy (Fig 1). Table 1 presents participants’ demographic characteristics. Comparison of participants’ characteristics showed statistically significant differences between the experimental and control groups in three variables (age of nurses, nurses’ and care workers’ experience of participating in end-of-life care workshops) (Table 1).

**Table 1:** Subjects’ demographic characteristics and comparison between groups.

		All	Experimental	Control
	Variables	Mean±SD or No (%)		
(a) Homecare nurse		64 (100%)	31 (100%)	33 (100%)
	Gender (Female)	61 (95.3%)	30 (96.8%)	31 (93.9%)
	Age (years)	47.1±8.6	49.3±8.3	45.1±8.5 *
	Years of clinical experience in the community	9.3±6.4	10.1±6.7	8.5±5.6
	Experience to support home death (number)	3.4±0.9	3.6±0.7	3.2±1.0
	Experience to participate in EOL care workshops	44 (68.8%)	28 (90.8%)	16 (48.0%) *
(b) Care Manager		129 (100%)	72 (100%)	57 (100%)
	Gender (Female)	106 (82.2%)	61 (84.7%)	45 (78.9%)
	Age (years)	52.9±9.5	51.4±8.8	54.5±10.0
	Years of clinical experience in the community	10.3±6.1	10.1±5.2	10.5±7.1
	Experience to support home death (number)	2.8±1.0	2.9±0.8	2.6±1.1
	Experience to participate in EOL care workshops	62 (48.1%)	35 (38.6%)	27 (47.4%)
(c) Head of Home Helper		98 (100%)	53 (100%)	45 (100%)
	Gender (Female)	83 (84.7%)	46 (86.8%)	37 (82.2%)
	Age (years)	51.8±10.8	52.8±9.3	50.8±12.3
	Years of clinical experience in the community	10.9±5.6	11.2±5.1	10.5±6.1
	Experience to support home death (number)	2.6±1.0	2.4±1.0	2.7±1.1
	Experience to participate in EOL care workshops	38 (38.8%)	25 (47.2%)	13 (28.9%) *

SD = standard deviation. EOL: End of Life

*There is statistically significant difference between the groups (experimental and control) using t-test, chi-squared test, or Mann-Whitney U test.

### Effect of intervention on confidence of and satisfaction with inter-professional collaboration

Before performing multivariate regression analyses, we confirmed that the exploratory factor analysis (maximum-likelihood method, promax rotation) of the FCCQ results among the participants produced seven factors, as identified in previous studies [21]. Further, the seven subscales were internally consistent: the Cronbach alpha values for the subscales were 0.81, 0.84, 0.88, 0.91, 0.92, 0.91, and 0.83. The multivariate regression analyses (controlled for baseline value, age, experience to participate in EOL care workshops as covariates) revealed significant between-group differences in confidence of interactions within professionals on all of the seven FCCQ subscales (Factor 1 (I can smoothly communicate with medical and welfare workers in other facilities) p = .044; Factor 2 (I understand other occupational roles of people working in the community) p < .0001; Factor 3 (I know the face, name, and characteristics of people associated with home care in the community) p = .003; Factor 4 (I have an opportunity to discuss matters with other healthcare workers in the community) p = .002; Factor 5 (I am connected to community care networks) p < .001; Factor 6 (I specifically understand community resources) p = .003; Factor 7 (A good network is maintained between the hospital and community, such as having a conference before hospital discharge) p < .0001) (Table 2).

**Table 2:** Effects of collaborative end-of-life intervention on face-to-face cooperative confidence ^a^ and job satisfaction ^b^ among home healthcare professionals between groups.

Outcome (range)		Time		Change from baseline	Difference between groups	
		T1	T2	T2-T1	Difference (95%CI)	p-value
		mean (SD)				
I. I can smoothly communicate with medical and welfare workers in other facilities (3–15^a^)						
	Experimental	11.5 (2.7)	11.8(2.3)	0.3(2.7)		
	Control	11.2 (3.0)	11.2(2.5)	0.0(2.7)	0.52 (0.01–1.02)	0.044
II. I understand other occupational roles of people working in the community (3–15^a^)						
	Experimental	10.0(2.4)	10.7(1.8)	0.7(2.1)		
	Control	9.4(2.7)	9.6(2.3)	0.2(2.5)	0.94 (0.50–1.37)	<0.001
III. I know the face, name, and characteristics of people associated with home care in the community (3–15^a^)						
	Experimental	8.8(2.7)	9.4(2.2)	0.6(2.5)		
	Control	8.5(2.9)	8.7(2.2)	0.2(3.2)	0.77 (0.26–1.29)	0.003
IV. I have an opportunity to discuss matters with other healthcare workers in the community (3–15^a^						
	Experimental	10.1(3.4)	10.7(2.6)	0.6(2.9)		
	Control	9.5(3.3)	9.7(2.2)	0.2(2.7)	0.80 (0.29–1.31)	0.002
V. I am connected to community care networks (3–15^a^)						
	Experimental	11.7(2.9)	11.9(2.2)	0.2(2.5)		
	Control	11.0(3.3)	10.8(2.5)	-0.2(2.4)	0.97 (0.49–1.45)	<0.001
VI. I specifically understand community resources (3–15^a^)						
	Experimental	10.9(2.8)	11.4(2.3)	0.5(2.4)		
	Control	10.4(3.0)	10.5(2.3)	0.1(2.5)	0.68 (0.23–1.12)	0.003
VII. A good network is maintained between the hospital and community, such as having a conference before hospital discharge (3–15^a^)						
	Experimental	11.7(2.6)	11.7(1.9)	0.0(2.5)		
	Control	11.0(2.7)	10.6(2.2)	-0.4(2.5)	0.80 (0.34–1.26)	<0.001
Job satisfaction (12–60^b^)						
	Experimental	43.0(5.3)	44.1(6.3)	1.1(4.9)		
	Control	43.0(5.9)	43.1(6.1)	0.1(4.4)	0.84 (-0.54–2.23)	0.231

T1:at baseline; T2: 7-month after the baseline

* We performed multivariate regression analyses for change from baseline with group, baseline value, age, experience to participate in EOL care workshops as covariates.

^a^ measured by a Face-to-face Cooperative Confidence Questionnaire (FCCQ), higher scores indicating more good collaboration

^b^ measured by Intrinsic Dimension of Minnesota Satisfaction Questionnaire (MSQ) (12 questionnaires)

By contrast, the MSQ scores showed no statistically significant between-group differences (p = .51) (Table 2).

## Discussion

Using a cluster-randomized trial, we investigated the effects of a collaborative end-of-life intervention program for home care nurses, care managers, and care workers. This intervention increased all aspects of home care professionals’ face-to-face cooperative confidence. To our knowledge, this is the first study to investigate the effects of an inter-professional collaborative education program among health- and social-care professionals. This suggests that the multidisciplinary end-of-life intervention program enhanced participants’ confidence in collaboration to support end-of-life patients. These aspects were: communicating seamlessly with medical and social care professionals from other facilities; knowing the faces and characteristics of other professionals; having opportunity to discuss matters with other professionals; connecting to the community care network; understanding community resources; and keeping good networks with hospitals.

Previous reports noted that care workers (who do not have medical licenses) often experience difficulties or gaps in providing end-of-life care, such as lack of knowledge and problems accessing and collaborating with medical professionals [31, 32]. This group of professionals also perceived various barriers, including concerns about the accessibility and accuracy of information, and discomfort engaging in end-of-life care [33, 34]. Recent reviews have suggested various solutions to these barriers. For example, strengthening collaboration between medical professionals and healthcare support workers (called social care professionals in Japan), providing training/education to promote and protect healthcare support workers, and supporting communication between team members [35–41]. We believe that the findings of this study may support the importance of training in education and communication as suggested by these reviews [35–41].

In terms of the effect of the intervention on job satisfaction, MSQ total scores (measuring participants’ intrinsic satisfaction) did not reveal significant between-group differences. Previous studies have shown that the development of professional identity may enhance professionals’ job satisfaction [42,43]. Professional identity is defined as attitudes, values, knowledge, beliefs, and skills shared by a professional group in the workplace [44,45]. Health-care professionals with a strong professional identity can provide effective services and high-quality patient care, develop competency in clinical expertise, and contribute to patient satisfaction [46,47]. Inter-professional role learning by using a case-based teamwork approach is effective for enhancing the awareness of team members contributing to client care [48]. The present study was unable to confirm that the intervention program contributed to participants’ understanding of other professionals and reinforcement of inter-professional identity; however, a previous investigation did find evidence for such an effect [49]. Further research is needed to clarify such interpretations about enhancing inter-professional identity. We intend to conduct additional study into the effect of an inter-professional collaborative program on job satisfaction among health- and social-care professionals.

The generalizability of our findings is uncertain. First, there were some implicit biases of the intervention effects in this study between experimental and control groups. The blinding of participants is not possible in this kind of behavior-approached intervention. Further studies are needed to resolve these limitations. Second, we recruited participants from one city and the number of subjects was relatively small. Third, there were some demographic differences between the experimental and control groups (age and experience of participating in end-of-life workshops). However, we analyzed the effect of the intervention by entering these variables as covariates. Fourth, this study could not measure patient outcomes and therefore could not analyze the correlation between professionals’ confidence and the end-of-life care actually provided. Further studies are needed to address these limitations. However, to our knowledge, this is the first study that used a randomized design to assess the impact of a multidisciplinary collaborative intervention program for health and social care workers in supporting end-of-life patients in the home care setting, as indicated a previous review [40]. In future research, we hope that this program could be applied with more home care professionals, to support them in managing end-of-life patients at home with better cross-professional collaboration.

## Supporting information

S1 Appendix Contents of a Face-to-face Cooperative Confidence Questionnaire (FCCQ) (prototype) a. (DOC)
