# Supplementary material for: Effect of a multidisciplinary end-of-life educational intervention on health and social care professionals: A cluster randomized controlled trial
Source: PLoS One. 2019 Aug 19;14(8):e0219589. doi: 10.1371/journal.pone.0219589 (PMC6699737; doi:10.1371/journal.pone.0219589)
Supplement: S1 Appendix — (DOC) [file pone.0219589.s001.doc]

Appendix. Contents of a Face-to-face Cooperative Confidence Questionnaire (FCCQ) (prototype) a

*[Factor1: I can smoothly communicate with medical and social care professionals in other facilities]**

I can easily ask about the patient to people working at other facilities*

I don’t have to hesitate when contacting people involved in the care of the patient

I understand what is the best way and when is the best time to contact people working at other facilities about the patient

*[Factor2: I understand other occupational roles of people working in the community]*

I roughly understand the problems arising in other jobs associated with home care in the community

I fully understand the actual work of people involved in home care

I roughly understand the general roles of health care workers involved in home care

*[Factor3: I know the face, name, and characteristics of people associated with home care in the community]*

I know the way of thinking of people associated with home care in the community

I understand the principles and current status of the facility associated with home care in the community

I know the characteristics of and how to deal with people associated with home care in the community

*[Factor4: I have an opportunity to discuss matters with other health care workers in the community]*

I have an opportunity to meet and talk with other health care workers involved in home care

I have an opportunity to gain new perspectives and acquaintances through contacting other health care workers with whom I have little contact in everyday life

I have an opportunity to share and discuss problems and difficulties regarding community networks for home care patients

*[Factor5: I am connected to community care networks]*

I have someone to ask about a patient

I know the best person to ask about patients in the community

I have someone to call when I have a problem regarding a patient

*[Factor6: I specifically understand community resources]*

I roughly understand long-term care services that can be used for patients

I understand the community medical resources and services that can be used for patients

I can specifically explain to patients and their family members about the community medical resources and services that can be used for patients

*[Factor7: A good network is maintained between the hospital and community, such as having a conference before hospital discharge]*

Upon discharge and admission, I discuss and provide necessary information for those involved in the patient’s care

A conference is conducted before hospital discharge to share information on the patient when transferring to home care

We decide on coping strategies for sudden changes in the course of the disease and a place to contact

a: cited by the manuscript “Fukui S. A measure to quantify the face-to-face cooperation level among home health care providers. Jpn Acad Home Care Physicians. 2014; 16 (1): 5–11 (in Japanese)”.

*All of 7 factors are consisted of 3 questionnaires each of which is evaluated from 1 (do not agree) to 5 (agree)
